# Recent advances of responsive scaffolds in bone tissue engineering

**DOI:** 10.3389/fbioe.2023.1296881

**Published:** 2023-11-17

**Authors:** Tongyu Zhu, Hongbo Zhou, Xiaojing Chen, Yuanjing Zhu

**Affiliations:** Hunan Clinical Research Center of Oral Major Diseases and Oral Health, Xiangya Stomatological Hospital, Xiangya School of Stomatology, Central South University, Changsha, Hunan, China

**Keywords:** responsive scaffolds, bone tissue engineering, stimuli, inflammatory, targeted drug delivery

## Abstract

The investigation of bone defect repair has been a significant focus in clinical research. The gradual progress and utilization of different scaffolds for bone repair have been facilitated by advancements in material science and tissue engineering. In recent times, the attainment of precise regulation and targeted drug release has emerged as a crucial concern in bone tissue engineering. As a result, we present a comprehensive review of recent developments in responsive scaffolds pertaining to the field of bone defect repair. The objective of this review is to provide a comprehensive summary and forecast of prospects, thereby contributing novel insights to the field of bone defect repair.

## 1 Introduction

Bone tissue defects present a significant health risk to individuals (Buza and Einhorn, 2016). Approximately four million surgical procedures are performed annually to address bone loss, utilizing grafts and/or substitutes, thereby establishing it as the second most commonly transplanted tissue worldwide (Greenwald et al., 2001). Although bone grafting serves as the preferred method for repairing extensive defects resulting from congenital anomalies, tumor removal, and traumatic fractures, it is accompanied by challenges such as limited availability, morbidity at the donor site, and inflammation, among others (Brydone et al., 2010; Tang et al., 2016).

The primary objective of bone tissue engineering is to develop bone-graft substitutes that can overcome the limitations associated with natural bone grafts (Shrivats et al., 2014). Scaffolds, which serve as a potential approach for treating bone defects, are currently being explored. The selection of appropriate biomaterials is of utmost importance in the fabrication of these scaffolds, and various techniques and materials are under investigation. The ideal bone graft substitutes should possess biocompatibility, biodegradability, and ease of production. Additionally, they should facilitate cell infiltration, stimulate bone growth, and provide biomechanical support during the regeneration of bone by osteoblasts (Bose et al., 2012).

Researchers have employed diverse scaffold materials to facilitate endogenous regeneration. Conventional scaffold materials comprise organic polymers such as collagen and hyaluronic acid, artificial polymers like polylactic acid, and biologically active inorganic materials like calcium phosphate, which enhance bone regeneration (Khan et al., 2008; Collins et al., 2021). However, conventional scaffolds exhibit a deficiency in controlled release capabilities, rendering them incapable of effectively regulating chronic inflammation as required (Ye et al., 2022). As a result, scholars have redirected their focus towards the advancement of responsive scaffolds.

Responsive scaffolds are considered to be groundbreaking in the field of bone repair. These scaffolds are comprised of materials that possess the capability to be activated and respond to various external stimuli, such as light, magnetism, and pH, or internal stimuli, including cytokines, enzymes, and biological signals. Responsive scaffolds demonstrate the ability to react to triggers originating from external regulatory equipment and internal microenvironment alterations, thereby enabling them to deliver drugs in a timely manner in response to a diverse array of circumstances (Wei et al., 2022). Moreover, they exhibit the capacity to react to both external and internal triggers, enabling them to deliver drugs as needed in response to a wide range of situations.

The occurrence of bone loss can lead to injury in both hard and soft tissues, with the microenvironment of diseased tissue exhibiting notable distinctions from that of healthy tissue. These disparities are believed to exploit various stimuli, including lower pH levels, elevated concentrations of reactive oxygen species (ROS), and heightened enzyme and osteoclast activities, thereby promoting bone resorption. By utilizing a responsive scaffold, it becomes possible to activate and target these specific stimuli, facilitating the precise delivery of drugs to modify the microenvironment and effectively repair the injury. For instance, researchers have developed a modified-scaffold composed of an electrospun asymmetric double-layer membrane made of polycaprolactone and collagen (PCL/Col) to address the low pH environment in bone defect sites. This composite scaffold exhibited the release of approximately 93% of Zn^2+^ ions from the PCL/Col/ZIF-8 membrane within 12 h under acidic conditions (pH 5.5). The pH-sensitive structure of the scaffold provides a favorable environment for the proliferation of osteoblasts, thereby presenting a promising approach for bone regeneration (Xue et al., 2021). Responsive scaffolds have demonstrated potential and approval in the treatment of bone injuries.

This review primarily examines the recent advancements in responsive biomaterials and scaffolds utilized in bone tissue engineering. It specifically delves into their application, material selection, scaffold design, and their efficacy in addressing bone defects. Furthermore, the review explores the current limitations and potential prospects for bone defect restoration, drawing upon substantial evidence that substantiates the favorable outcomes achieved through the implementation of functionalized responsive scaffolds.

## 2 The categories of responsive scaffolds

**FIGURE 1:**
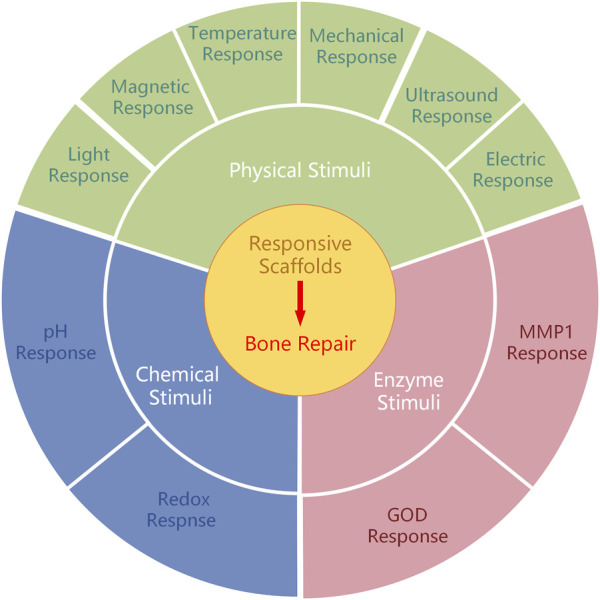
Different types of responsive scaffolds for bone repair.

This study primarily encompasses three primary categorizations of stimulus-responsive scaffolds based on the source of stimuli: physical stimuli (e.g., light, temperature change, electric field, magnet, and ultrasound), chemical stimuli (e.g., pH level and ROS), and enzyme stimuli (Figure 1) (Table 1).

**TABLE 1:** The summarization of recent responsive scaffolds.

Scaffold categories	Specific scaffold material	Growth factors or drugs	Animal model/*in vitro* study	Function	Ref
temperature-responsive	elastin-like polymer (VPAVG)_220_	bone morphogenetic protein-2 (BMP-2) and bone morphogenetic protein-14 (BMP-14)	C2C12 cells	induced osteogenic mineralization	Bessa et al. (2010)
temperature-responsive	poly(ε-caprolactone-co-lactide)-b-PEG-b-poly(ε-caprolactone-co-lactide) (PCLA) and O-phosphorylethanolamine	bone morphogenetic protein 2 (BMP-2)	subcutaneous administration into the dorsal region of Sprague-Dawley (SD) rats	biomineralized *in situ*	Kim et al. (2020)
temperature-responsive	chitosan (CS) and methylcellulose (MC)	veratric acid (VA)	mouse mesenchymal stem cells	promoted osteogenic differentiation	Durairaj et al. (2023)
temperature-responsive	hydroxyapatite (HA), Gelatin (GN) and Fe_3_O_4_	ibuprofen (IBU)	MTT assay within the cell environment	highly biocompatible	Sahmani et al. (2020)
light-responsive	strontium and ibuprofen-loaded black phosphorus (BP + IBU@SA microspheres) into aminated modified poly-L-lactic acid (PLLA)	ibuprofen (IBU)	MC3T3-E1 cells	improved cell adhesion and proliferation and induced apatite formation	Chen et al. (2021)
light-responsive	thin-film silicon (Si) embedded into hydroxyapatite mineralized collagen/poly(ε-caprolactone) (PLA) structures	-	5 mm-sized SD rat circular bone defect model	improved osteogenesis	Wang et al. (2023)
electric-responsive	poly (l-lactic acid)-block-aniline pentamer-block-poly (l-lactic acid) (PLA-AP) with poly (lactic-co-glycolic acid)/hydroxyapatite (PLGA/HA)	human bone morphogenetic protein-4 (hBMP-4)	rabbit radial defect model	improved cell proliferation ability, enhanced osteogenesis differentiation and bone healing	Cui et al. (2020)
electric-responsive	gelatin-graft-poly-pyrrole	H_2_O_2_	-	sustained oxygen release	Nejati et al. (2020)
electric-responsive	silicon dioxide with poly(dimethylsiloxane) (SiO_2_/PDMS)	-	5 mm-sized SD rat circular bone defect model	facilitated bone regeneration	Qiao et al. (2022)
mechanical-responsive	hydroxyapatite/barium titanate (HA/BT)	-	MTT assay within L929 cells	highly biocompatible	Zhang et al. (2014)
magnetic-responsive	poly (vinylidene fluoride) (PVDF), and magnetostrictive particles of CoFe_2_O_4_	-	MC3T3-E1 cells	promoted preosteoblasts proliferation	Fernandes et al. (2019)
magnetic-responsive	polycaprolactone (PCL) microparticles, encapsulating magnetic nanoparticles (MNPs)	placental proteins	umbilical cord mesenchymal stem cells (UC-MSCs)	promoted osteogenic differentiation	Lanier et al. (2021)
ultrasound-responsive	polylactic acids (PLA) embedded in alginate hydrogels	stromal cell-derived factor-1 (SDF-1) and bone morphogenetic protein 2 (BMP-2)	SD rats femoral bone defect model	repaired bone defect *in situ*	He et al. (2023)
pH-responsive	polycaprolactone/collagen (PCL/Col) membrane modified by zeolitic imidazolate framework-8 (ZIF-8)	-	SD rats calvarial defect model	increased osteoinductivity along with blood vessel formation	Xue et al. (2021)
pH-responsive	chitosan loaded with ZIF-8	vancomycin (VAN)	MC3T3-E1 cells	promoted high proliferation and osteogenic activities	Karakeçili et al. (2019)
ROS-responsive	LBL-compatible poly (thioketal β-amino amide) (PTK-BAA) polycation	bone morphogenetic protein 2 (BMP-2)	8 mm-sized SD rat circular bone defect model	increased new bone formation	Martin et al. (2021)
enzyme-responsive	KLDL-MMP1 (Ac-KLDLKLDLVPMSMRGGKLDLKLDL-CONH2) peptides	bone marrow mesenchymal stromal cell-derived exosomes (BMSC-Exos)	6 mm-sized SD rat circular bone defect model	recruited stem cells and promoted osteodifferentiation in response to neovascularization and accelerate tissue regeneration	Yang et al. (2023)
enzyme-responsive	polycaprolactone/chitosan nanofibers with glucose oxidase (GOD)	dexamethasone (DEX)	MC3T3-E1 cells	promoted MC3T3-E1 cells' osteogenic differentiation in high-glucose environments	Jia et al. (2023)

### 2.1 Physical stimuli

Physical-responsive scaffolds are predominantly comprised of materials that are sensitive to physical stimuli. These materials possess a structure that can be reconfigured when exposed to various factors, including light, magnetism, temperature, ultrasonic waves, and magnetic fields. Consequently, these alterations in structure facilitate the delivery of drugs. For instance, temperature-responsive scaffolds exhibit stability in healthy tissue, but undergo degradation in diseased tissue. In the initial phases of bone defects, inflammation induces a localized increase in temperature. This change in temperature can serve as an endogenous stimulus for the scaffold to respond and subsequently release the drug (Zhang et al., 2013; Karimi et al., 2016b).

Under specific circumstances, elastin-like polypeptides (ELPs) exhibit a lower critical solution temperature (LCST) in contrast to other synthetic polymers (Tamburro et al., 2005). Upon surpassing this transition temperature, ELPs undergo a first-order phase transition, resulting in the formation of a peptide and water-rich phase (Krishna et al., 2012; Zhao et al., 2016). This distinctive characteristic has sparked considerable enthusiasm in the advancement of biomaterials capable of reacting to external stimuli. In a recent study, researchers have successfully synthesized elastin-like self-assembly nanoparticles with thermos-responsive characteristics. These nanoparticles were employed for the controlled release of bone morphogenetic protein-2 (BMP-2) and bone morphogenetic protein-14 (BMP-14), exploiting the reverse temperature transition of bio-generated polymer(VPAVG) _220_ (Bessa et al., 2010). This distinctive property can be harnessed to mitigate inflammation and facilitate bone regeneration under specific conditions. In the field of bone tissue engineering, a wide range of physical-responsive scaffolds are frequently utilized to modulate drug delivery.

#### 2.1.1 Temperature response

Temperature-responsive scaffolds can undergo structural reconstruction and release drug payloads when the desired temperature is achieved, as they are triggered by changes in the surrounding environment. Thermo-responsive polymers, employed in the fabrication of these scaffolds, may experience phase transitions above the LCST (Bordat et al., 2019) or expedite degradation when exposed to elevated temperatures. For instance, certain thermo-responsive polymers undergo a transition from a liquid state to a stable viscoelastic gel, while certain thermo-sensitive polymers exhibit accelerated degradation at elevated temperatures, thereby facilitating the controlled release of drugs.

In a recent study, a cohort of alginate bioconjugates comprising micrografted poly(ε-caprolactone-co-lactide)-b-PEG-b-poly(ε-caprolactone-co-lactide) (PCLA) and o-phosphorylethanolamine were synthesized with the aim of facilitating bone regeneration (Kim et al., 2020). These bioconjugated salts have the ability to undergo a conversion into durable viscoelastic gels when administered *in vivo* and subjected to physiological temperature, surpassing the LCST threshold. This conversion enhances the mechanical characteristics and fosters bone regeneration, thereby suggesting their potential utility in promoting bone formation. Methylcellulose is a cellulose polysaccharide known for its biocompatibility, biodegradability, and hydrophilicity. It demonstrates gelation properties, resulting in gel formation at specific temperatures (Lioubavina-Hack et al., 2005; Kim et al., 2018). Composite hydrogels, comprising chitosan and methylcellulose, encapsulate veratric acid and exhibit desirable biocompatibility. These hydrogels gelatinize at 37°C, making them suitable for use as a restorative agent to enhance osteoblast differentiation (Durairaj et al., 2023).

Endogenous temperature stimulation is elicited by fluctuations in temperature conditions at the site of the lesion. The lesion tissue undergoes pathological deformation and hyperthermia, which stem from trauma and tumors, thereby triggering the release of inflammatory factors and evaluation of the local environmental temperature. Consequently, the scaffold structure in pathological sites undergoes alterations when the temperature is elevated. During the initial phase of bone defect, the localized temperature elevation can serve as a stimulus for scaffold response and/or drug release (Zhang et al., 2013; Karimi et al., 2016b).

Nanocarriers engineered for thermal responsiveness have the potential to maintain stability at the physiological temperature of the human body. Upon exposure to external heat or assessment of the local environmental temperature, these carriers can efficiently release therapeutic agents either promptly upon heating or in a controlled manner at the site of disease. In a recent study, Fe_3_O_4_ nanoparticles were employed by researchers within a magnetic field to induce heat generation and eradicate infected cells (Abdellahi et al., 2018a). The magnetite nanoparticles (MNPs) possess the ability to elevate temperature upon exposure to an alternating magnetic field, thus facilitating the degradation of the drug carrier for drug release (Abdellahi et al., 2018b; Sahmani et al., 2018). In this particular scenario, hydroxyapatite (HA) and gelatin (GN) were integrated with MNPs to fabricate bio-nanocomposite scaffolds, which subsequently underwent degradation under magnetothermal conditions. The results indicate that the prepared scaffold exhibits promising potential for utilization in bone tissue engineering for both biological and thermal applications (Sahmani et al., 2020).

The achievement of thermal specificity in temperature-responsive systems poses a significant obstacle due to the restricted variability observed in pathological tissues within living organisms. Consequently, future research endeavors should prioritize the development of scaffold materials that are more responsive to lower temperatures, possess enhanced stability in normal tissues, and ensure greater safety.

#### 2.1.2 Light response

Various light-responsive materials exhibit different responses to various wavelengths of light, thereby facilitating the identification of suitable materials for diverse clinical needs. Upon exposure to light, light-responsive scaffolds undergo changes in their physical properties, thereby enabling efficient drug delivery. This responsiveness of scaffolds is primarily attributed to the degradation of materials containing light-sensitive components or the modification of light-sensitive molecules. Consequently, when scaffolds are exposed to light, the drug bound or encapsulated within them is released (Mayer and Heckel, 2006; Chen and Zhao, 2018).

Black phosphorus (BP), a novel nanomaterial characterized by its two-dimensional framework, exhibits remarkable biosafety, inherent biocompatibility, and photosensitivity (Pandey et al., 2020). In a recent study, a multifunctional nanofiber scaffold was developed by incorporating ibuprofen black phosphorus (BP + IBU@SA microspheres) and sodium alginate microspheres onto aminated poly-L-lactic acid (PLLA) nanofibers. This scaffold demonstrates exceptional near-infrared light-responsive release capabilities and anti-inflammatory properties. BP was employed to induce the destruction of polymeric shells through the utilization of near-infrared (NIR)-mediated photothermal performance, thereby achieving controlled drug release. By subjecting the scaffolds to NIR light, the adverse effects of rapid drug release can be mitigated, while maintaining the drug concentration at an optimal level to meet the specific requirements of bone repair. The conducted investigations have demonstrated that the incorporation of functionalized scaffolds enhances cell adhesion, proliferation, and apatite formation, rendering it a viable and promising approach for bone tissue engineering (Chen et al., 2021).

**FIGURE 2:**
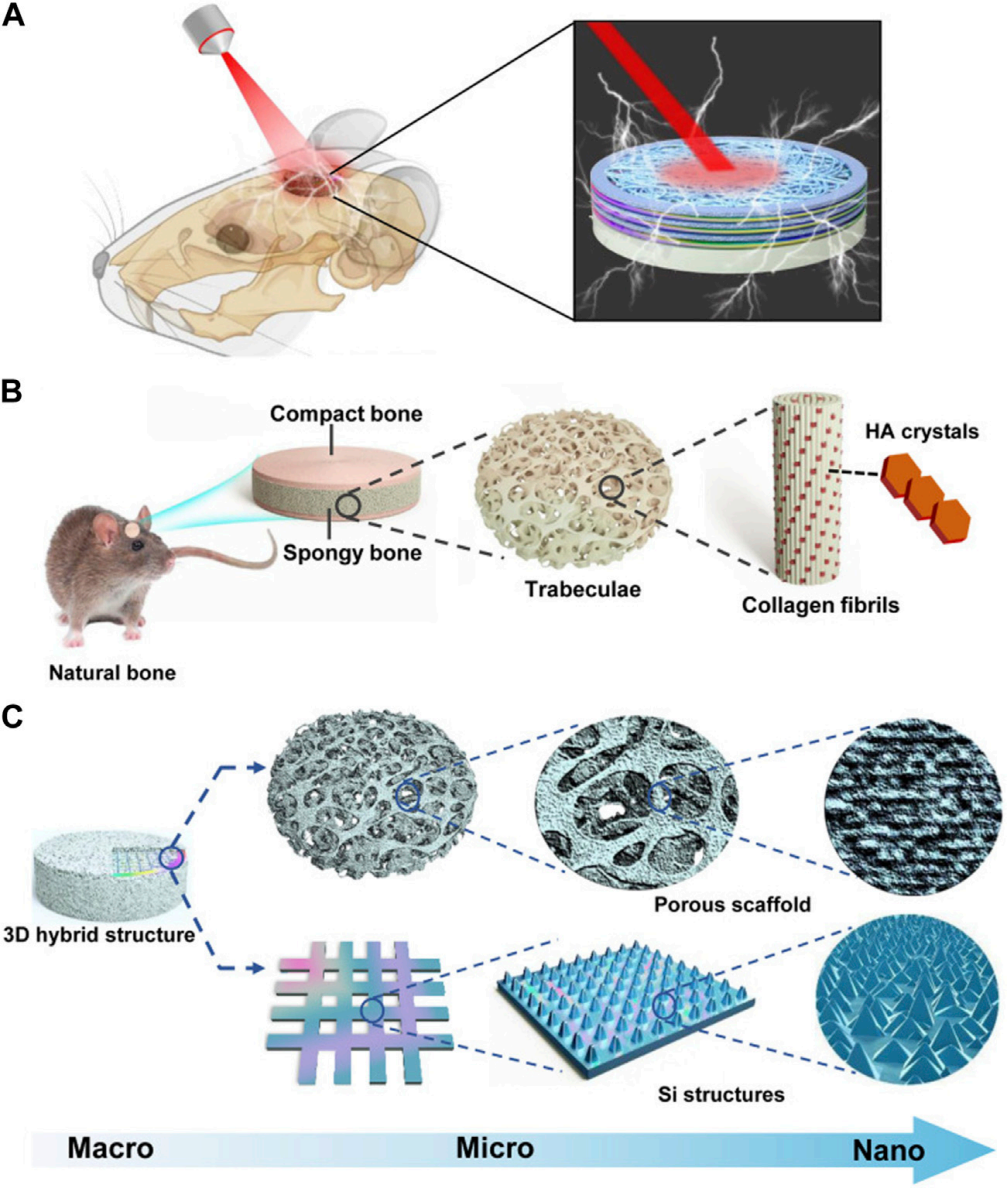
Bioregenerative 3D optoelectronic scaffold with Si nanostructures for bone regeneration. **(A)** An illustration of an implantable scaffold in concept. **(B)** Native bone hierarchical structure. **(C)** Structural design of the 3D hybrid scaffold. Reproduced from (Wang et al., 2023) with permission. Copyright 2023 AAAS.

Silicon (Si) is widely employed as a semiconductor material in bio-implantation devices (Kang et al., 2016; Parameswaran et al., 2018). When subjected to near-infrared illumination, Si structures produce electrical signals that depolarize cell potentials and trigger intracellular calcium activation. Consequently, these optoelectronic signals play a role in directing hBMSCs towards osteogenic differentiation (Wang et al., 2023). Recently, a three-dimensional (3D) biomimetic scaffold utilizing thin-film Si microstructures has been developed. Through the utilization of NIR light, researchers have discovered that the Si film facilitates the attachment and growth of cells. The Si-based hybrid scaffold offers a 3D hierarchical structure that effectively governs cell growth and regulates cell behavior via light-responsive electrical signals. These silicon structures are remotely manipulated by infrared radiation to regulate the depolarization of stem cell membranes, resulting in heightened Ca^2+^ activities for hBMSCs, as well as improved potential and intracellular calcium dynamics. Consequently, this process promotes both cell proliferation and differentiation. The utilization of silicone scaffolds resulted in enhanced bone formation when subjected to light stimulation (Wang et al., 2023) (Figure 2).

However, the advancement of light-responsive systems continues to encounter various obstacles. In numerous applications, the ability of radiation below 650 nm to penetrate tissue beyond a depth of 1 cm is limited, while NIR light within the range of 650–900 nm (as water absorbs wavelengths longer than 900 nm) can penetrate up to 10 cm. However, these penetration depths are not considered clinically significant due to being either too shallow or too deep *in vitro* (Weissleder, 2001; Fomina et al., 2012). Additionally, further quantitative investigation is necessary to evaluate the biological safety of light-sensitive materials and ascertain the optimal duration and intensity of light exposure.

#### 2.1.3 Electric response

Electrical stimulation (EStim) has undergone extensive research and has proven to be an effective intervention in medical settings for the purpose of enhancing bone healing (Bhavsar et al., 2020), as it exerts influence on the migration (Yuan et al., 2014), proliferation (Ercan and Webster, 2008), differentiation (Eischen-Loges et al., 2018) of bone cells. Presently, the integration of electrostimulation therapy with electric-responsive stents is regarded as a compelling approach in clinical practice (Palza et al., 2019; Vaca-González et al., 2019).

Endogenous electrical currents exert a substantial influence on diverse physiological processes in the human body. These naturally occurring electrical fields possess the capacity to induce either depolarization or hyperpolarization of the membrane potential in living tissues, thereby eliciting the activation of signaling factors that facilitate cell proliferation and migration, including those of bone cells (Funk, 2015). Exogenous electrical stimulation including alternating current (AC), which reverses direction periodically and direct current (DC) which flows in one direction both have effects on bone tissue and scaffold (Chen et al., 2013). Electric-responsive scaffolds possess inherent bioactivity and can facilitate tissue formation with or without the need for external electrical stimulation. These scaffolds are capable of responding to electrical fields in living tissues, thereby expediting drug release. Consequently, electric-responsive scaffolds have been employed in various studies within the field of bone tissue engineering.

Conductive polymers, namely polyaniline, poly-pyrrole, polythiophene, and their derivatives (Cui et al., 2012; Xie et al., 2015) have been found to augment cellular activities, including cell adhesion, proliferation, differentiation, migration, and protein secretion, at the interface between the polymer and tissue, regardless of electrical stimulation (Hardy et al., 2013). These polymers demonstrate favorable biocompatibility in both *in vivo* and *in vitro* settings, while also exhibiting high conductivity under physiological conditions. Polyaniline (PA) is a conductive polymer that exhibits the ability to undergo transference when subjected to pulsed EStim (Wang et al., 2017). Additionally, polylactide (PLA) is a polymer known for its favorable biodegradability (Huang et al., 2007). In light of these aforementioned attributes, a novel electric-responsive scaffold has been developed, comprising a main chain composed of poly (l-lactic acid)-block-aniline pentamer-block-poly (l-lactic acid) (PLA-AP) and a triblock copolymer of poly (lactic-co-glycolic acid)/hydroxyapatite (PLGA/HA). The composite scaffold (PLGA/HA/PLA-AP/phBMP-4) underwent degradation upon electrical stimulation (Cyclic voltammograms (CV), scanning rate of 100 mVs^−1^) to control the release of phBMP-4 and regulate gene expression of doxycycline (Dox). In an experimental model involving rabbit radius defects, the electric-responsive scaffold demonstrated enhanced cell proliferation, improved osteogenic differentiation, and influenced the process of bone healing (Cui et al., 2020).

Poly-pyrrole has garnered significant attention in academic research due to its exceptional conductivity. In this study, H_2_O_2_-loaded polylactic acid microparticles were manufactured, and gelatin-graft-poly-pyrrole with varying pyrrole contents and periodate-oxidized pectin were synthesized to create an injectable conductive hydrogel/microparticle scaffold. This scaffold demonstrated the ability to sustain oxygen release for a duration of 14 days. The conductivity of the scaffold can enhance the bone healing process when responding to electrical stimuli, making it a promising candidate for bone tissue engineering applications (Nejati et al., 2020).

**FIGURE 3:**
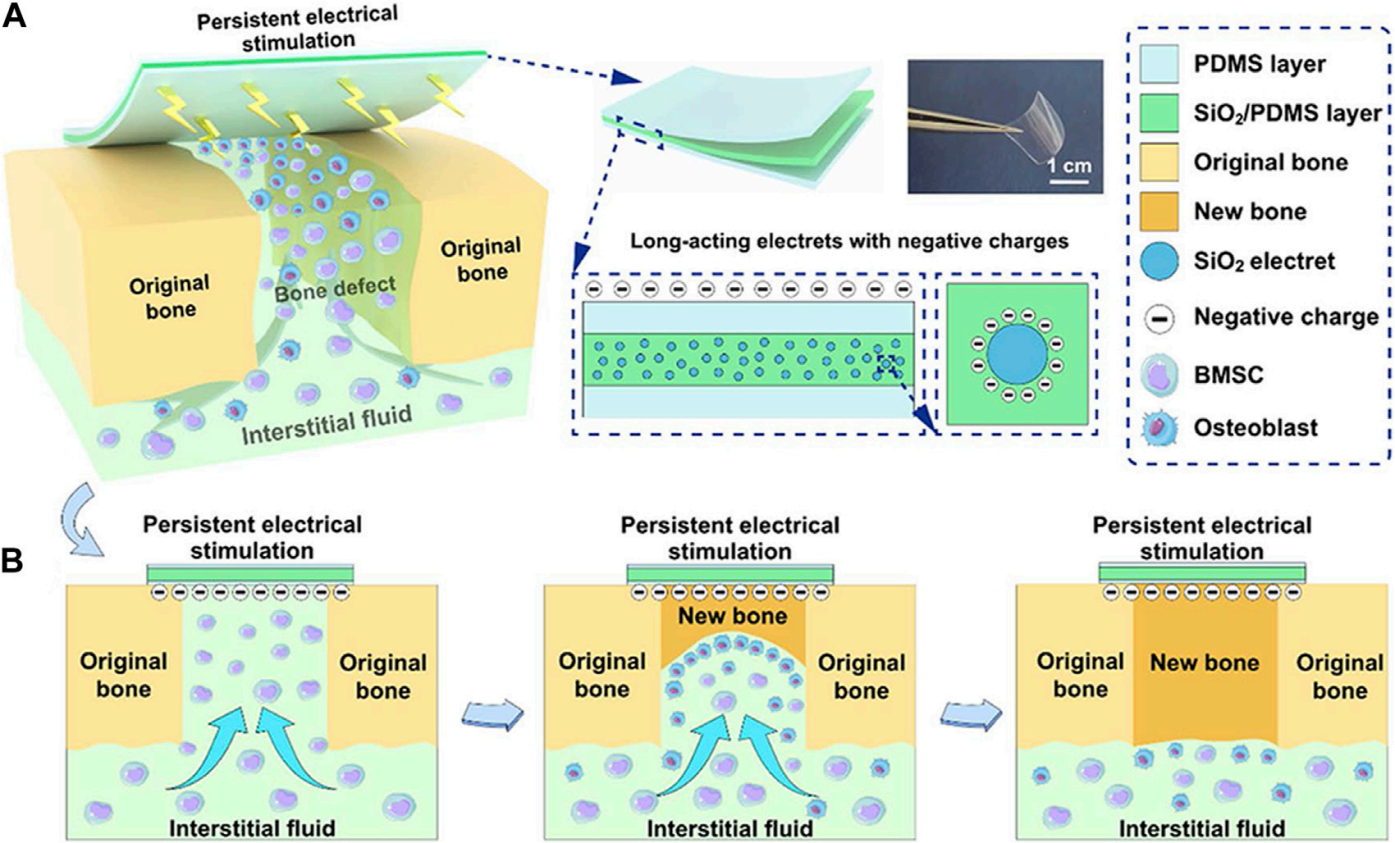
The schemes of electreted sandwich membranes. **(A)** Illustration of persistent electrical stimulation provided by electreted sandwich-like SiO_2_/PDMS composite membranes. **(B)** Implanted composite membranes act as native periosteum covering the bone defect region to enhance well-integrated bone formation and regeneration. Reproduced from (Qiao et al., 2022) with permission. Copyright 2022 American Chemical Society.

Electret materials, known for their enduring polarization properties (Zhang et al., 2023), have the ability to generate intrinsic electrical stimulation when subjected to an external electric field (Guo et al., 2022; Lin et al., 2022; Qiao et al., 2022). In tissue engineering, electret materials commonly employed include inorganic compounds like silicon dioxide (SiO_2_) (Qiao et al., 2022), zinc oxide (ZnO) (Zhu et al., 2018), HA (Nakamura et al., 2009), as well as biopolymers such as proteins (e.g., collagen), polysaccharides (e.g., chitin), and polynucleotides (e.g., DNA), also demonstrate the phenomenon of the electron effect (Zheng et al., 2020). SiO_2_, a material with electret properties, exhibits favorable biocompatibility and charge retention ability (Li et al., 2015). In order to enhance its electroactive properties, researchers developed a composite membrane by integrating silicon dioxide with poly(dimethylsiloxane) (SiO_2_/PDMS). The composite membranes underwent polarization through the application of an external electric field, resulting in the retention of residual charge for a duration of up to 6 weeks. The electreted SiO_2_/PDMS membranes demonstrated a favorable electrical microenvironment, leading to enhanced osteogenic differentiation of BMSCs *in vitro* and accelerated bone defect healing *in vivo* (Qiao et al., 2022) (Figure 3).

The potential application of electrical stimuli-responsive scaffolds in bone repair shows promise. However, the controllability of electric field changes in organisms remains uncertain, necessitating further investigation into the application of telephony stents.

#### 2.1.4 Mechanical response

Mechanical-responsive materials possess the ability to promptly alter their physiochemical attributes when subjected to mechanical force or deformation (Shabani and Bodaghi, 2023). Piezoelectric biomaterials are a class of intelligent materials capable of producing electrical activity in response to mechanical stimulation, independent of the need for external electrical devices ( Zheng et al., 2020 ). Piezoelectricity arises from the inherent crystal or chemical structure of materials, leading to the development of a net dipole or charge during mechanical deformation. Additionally, piezoelectric materials possess the ability to modulate cellular behavior by generating surface charges in response to deformation caused by cellular interaction. This characteristic offers novel avenues for biomechanical simulation, bone regeneration, and bone defect repair (Tandon et al., 2018; Khare et al., 2020).

Piezoelectric materials can be classified into various categories including polymers (such as PLLA and poly (vinylidene fluoride) (PVDF)), ceramics [such as HA and barium titanate (BT)], natural materials like collagen, and composite polymers. An example of such composites is the aligned porous BT/HA composites, which have been developed to possess high piezoelectric coefficients owing to their exceptional piezoelectric property. These composites serve as a charge supplier, thereby stimulating the bone healing process, and exhibit similar charge supply properties and stress-generated potentials as natural collagen bone (Baxter et al., 2010; Zhang et al., 2014).

Collagen, a naturally occurring protein and integral component of bone, exhibits piezoelectric properties that render it well-suited for tissue engineering applications. Specifically, the piezoelectric nature of collagen within bone induces the generation of a streaming potential when subjected to stress, leading to a decrease in hydraulic permeability and an augmentation in stiffness (Ahn and Grodzinsky, 2009; Ferreira et al., 2012). The suitability of the collagen-HA piezoelectric composite scaffold for cellular growth and bone healing has been demonstrated in previous research (Silva et al., 2001). Nevertheless, this scaffold is subject to certain limitations, including low mechanical stiffness, rapid degradation, and potential toxicity resulting from the use of crosslinking agents.

Despite facing challenges related to material stability, biocompatibility, and the need to balance mechanical properties, the investigation of piezoelectric materials in the realm of bone tissue engineering presents promising opportunities for the treatment of bone defects and the regeneration of bone.

#### 2.1.5 Magnetic response

The utilization of magnetic nanoparticles in bone tissue engineering has gained attention due to their inherent magnetism and the magnetocaloric effect, among other factors. These nanoparticles demonstrate a responsive characteristic towards magnetic fields, including both alternating magnetic field (AMF) that periodically change direction, and constant magnetic field (CMF) that remain in one direction (Goharkhah et al., 2015). Moreover, they possess the potential to augment the osteoinductive, osteoconductive, and angiogenic properties of scaffolds (Dasari et al., 2022).

**FIGURE 4:**
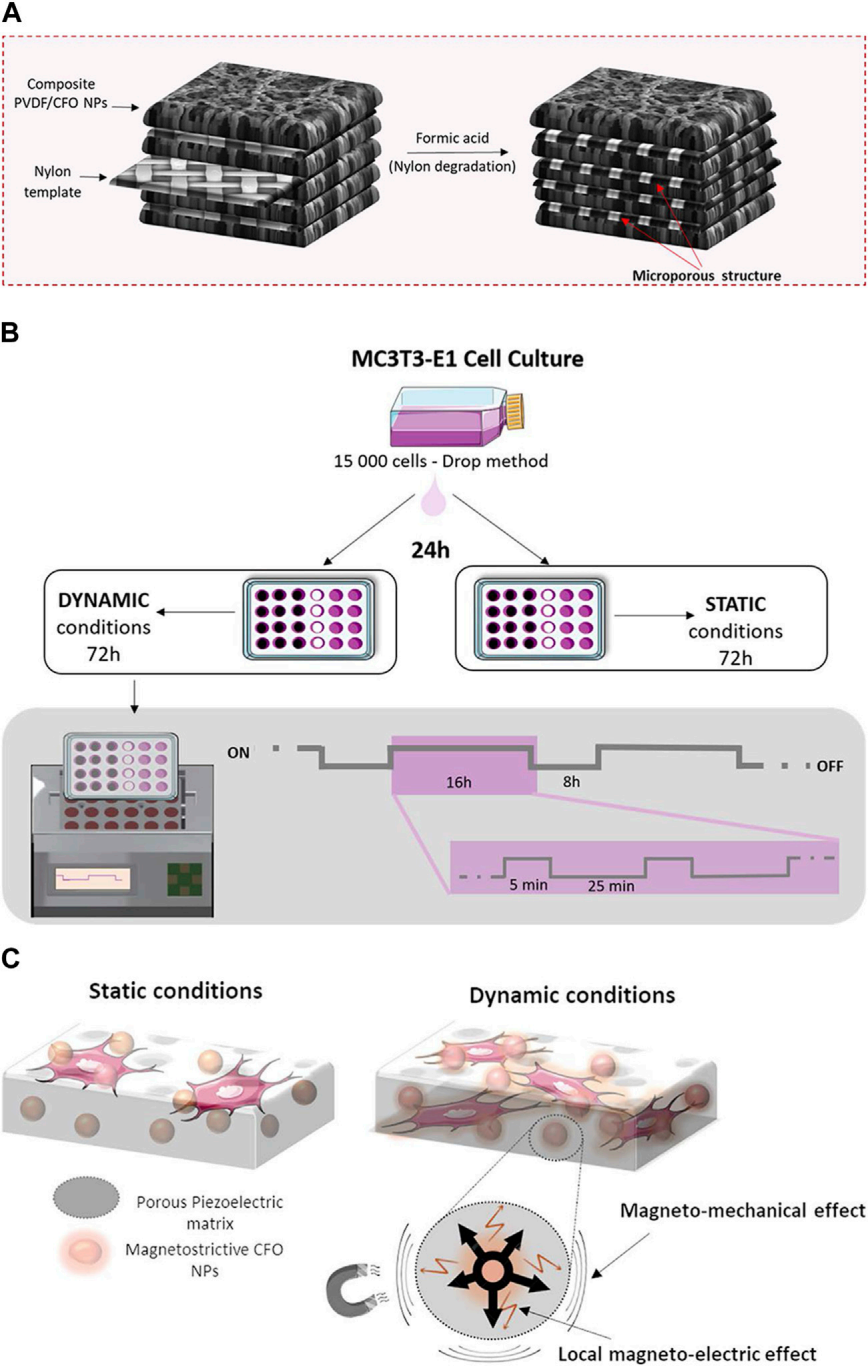
The schemes of 3D magnetoactive scaffolds for bone tissue engineering. **(A)** Schematic representation for the 3D scaffold development. **(B)** Schematic representation of the cell culture assays and stimulation profile. **(C)** Schematic representation of magnetomechanical and local magnetoelectrical properties of 3D scaffolds upon the magnetic stimuli. Reproduced from (Fernandes et al., 2019) with permission. Copyright 2019 American Chemical Society.

A magnetic-responsive scaffold comprising of a piezoelectric polymer, PVDF, and magnetostrictive particles of CoFe_2_O_4_ has been successfully fabricated, with nylon template structures utilized to facilitate the solvent casting process. The investigation revealed that the PVDF component of the scaffold undergoes crystallization into the electroactive β-phase when subjected to magnetic and/or electromagnetic stimulation (permanent magnets, frequency of 0.3 Hz), thereby enhancing the proliferation of preosteogenic cells. This observed phenomenon can be attributed to the interplay between the magnetic and electromagnetic properties of the magnetic nanoparticles upon stimulation (Fernandes et al., 2019) (Figure 4). The magnetomechanical and magnetoelectric response of the scaffolds is believed to be a valuable resource.

In a separate investigation, the magnetic-responsive scaffold is comprised of PCL microparticles that enclose MNPs and placental proteins. The MNPs, due to their magnetocaloric effect, induce heating and subsequent melting of the PCL upon exposure to AMF (strength from−1 to 1 T), thereby facilitating the diffusion of proteins from the microparticles to stimulate bone formation. Upon deactivation of the magnetic field, the PCL solidifies once again, potentially enabling repeated administration of drugs in a cyclic manner (Lanier et al., 2021). This present study introduces a magnetic-responsive delivery system designed for localized drug release, with potential applications in bone regeneration.

The magnetic field presents a superior setting for external stimuli-responsiveness in comparison to light and temperature due to its ability to fully penetrate human tissue and initiate release, while also allowing for complete external control. Nevertheless, magnetic nanoparticle could face drawbacks of diffusion out within one or 2 days, thus preventing a continuous release (Veres et al., 2022), during the bone defect treatment and Iron oxide magnetic nanoparticles may mediate ROS generation and have an impact on other cells (Hohnholt et al., 2011; Sruthi et al., 2018).

#### 2.1.6 Ultrasound response

Ultrasound, a mechanical wave with a high frequency (≥20 kHz), possesses the ability to be concentrated and transmitted within a particular medium, thereby finding utility in various clinical domains including *in vivo* imaging and physical therapy (Wheatley and Cochran, 2013). Furthermore, ultrasound exhibits potential in addressing bone defects as it can influence the biological aspects and drug administration characteristics of materials (Wei et al., 2021; He et al., 2023).

In order to obtain biomimetic scaffold composites (BSCs), researchers fabricated acoustically responsive hydrogel scaffolds (ARSs) that were developed and incorporated with stromal cell derived factor-1 (SDF-1) and BMP-2. The alginate hydrogel scaffold was degraded through pulsed ultrasound (p-US) irradiation, resulting in the exposure of ARSs to BMSCs due to its thermal effect. Subsequently, sinusoidal continuous wave ultrasound (s-US) irradiation was applied to stimulate the intrinsic resonance of ARSs, thereby facilitating the capture of endogenous BMSCs on the scaffolds and significantly enhancing their adhesion and growth for the *in situ* repair of bone defects (He et al., 2023).

The ultrasound-responsive scaffold facilitates the precise release of drugs and recruitment of cells in a spatiotemporal manner, while minimizing adverse effects through a non-toxic pathway (Pitt et al., 2004; Zardad et al., 2016). Nonetheless, the uncontrolled depth of ultrasound penetration and the potential thermal effect necessitate further investigation. In contrast to ultrasound, shockwave, which is a prevalent mechanical wave, exhibits greater shock amplitude and energy (Smallcomb et al., 2022). It is commonly employed to facilitate the biological healing processes of bones. Although shockwave is seldom reported as a stimulus source for responsive scaffolds, it offers a promising and innovative avenue for the repair of bone defects (Cheng and Wang, 2015).

### 2.2 Chemical stimuli

Chemically-responsive scaffolds are primarily constructed using materials that demonstrate sensitivity to specific variations in environmental concentration. When exposed to changes in the pH value, ROS concentration, ion concentration, and other conditions, the drug-encapsulated scaffold undergoes stimulation, leading to the rupture of responsive chemical bonds or modification of the functional group structure within the scaffold. Consequently, this process triggers the release of the drug. For example, ROS concentration can be activated by phagocytes (such as granulocytes and macrophages) under inflammatory conditions after trauma, and phenylborate pinanol ester (PBAP), a compound that can be combined on scaffold, break chemical bonds and fracture under high ROS, which lead to the quickly drug releasing on the certain site (Zhang et al., 2017; Yuan et al., 2021). Therefore, the development of chemical-responsive materials holds potential in facilitating targeted drug delivery at the site of injury to promote bone repair. Here are several common types of chemical-responsive scaffolds on bone regeneration.

#### 2.2.1 pH response

pH-responsive scaffolds are specifically engineered to react to alterations in pH levels, which are induced by the release of inflammatory factors from injured tissues. Extensive research has demonstrated that the pH value can decrease to 6.5 within a span of 60 h following the onset of inflammation (Caliceti, 2011). Furthermore, various organelles exhibit distinct pH values, such as lysosomes (4.5–5), endosomes (5.5–6), golgi apparatus (6.4), and cytosol (7.4) (Karimi et al., 2016a). Consequently, it becomes feasible to incorporate pH-responsive chemical groups into scaffold materials, thereby empowering the scaffold to regulate the release of drugs within the affected tissue.

Zeolitic imidazolate framework-8 (ZIF-8) belongs to the class of metal-organic frameworks (MOFs), which are formed through the connection of metal ions or clusters with organic ligands. Its remarkable pH-sensitivity has led to its application as a bone substitute and drug carrier (Zheng et al., 2016). Research has demonstrated that ZIF-8 is capable of releasing Zn^2+^ ions in acidic environments, thereby displaying a favorable osteogenic impact (Liu et al., 2022). To facilitate the promotion of vascularized bone regeneration, electrospun polycaprolactone/collagen (PCL/Col) membranes were modified with ZIF-8. The ZIF-8 structure experienced collapse and subsequent release of Zn^2+^ ions at a pH value of 5.5. Within a 12-h timeframe, approximately 93% of Zn^2+^ ions were discharged from the PCL/Col/ZIF-8 composite membrane under acidic conditions (pH 5.5). Utilizing this pH-responsive scaffold, concurrent restoration of blood vessels and bone was achieved in a rat model with calvarial defects (Xue et al., 2021).

In a separate study, ZIF-8 nanocrystals were employed as a carrier for vancomycin in order to achieve a delivery profile that responds to changes in pH. These nanocrystals were incorporated into chitosan fiber-scaffolds to create a potential substitute for bone tissue, which also possessed antimicrobial properties and facilitated interaction with osteoblast cells. Following a 48-h period at a pH of 5.4, the release of vancomycin reached a plateau at 77%, subsequent to the increased dissolution of ZIF-8 under acidic conditions. This dissolution served to diminish the activity of *S. aureus* and promote the differentiation of preosteoblasts into osteoblasts (García-González et al., 2018; Karakeçili et al., 2019).

The pH-responsive system presents a captivating approach for drug delivery, leveraging the pH discrepancies observed in various tissues within the living organism. Nevertheless, the exclusive reliance on pH reaction systems may encounter limitations in terms of specificity and sensitivity, given the inconsistent magnitude of pH disparity between the target tissue and healthy tissue.

#### 2.2.2 Redox response

ROS, encompassing highly reactive ions, free radicals or molecular compounds such as superoxide (O^2-^), hydroxyl radicals (·OH), hypochlorite ion (ClO^−^), and hydrogen peroxide (H_2_O_2_), play a significant role as signaling molecules in the progression of inflammatory disorders. Given their close relationship with bone growth and remodeling, ROS hold particular appeal for augmenting material responsiveness (Martin et al., 2021).

At the site of a bone defect caused by inflammation, polymorphonuclear neutrophils (PMNs) produce an excessive amount of ROS, leading to endothelial dysfunction and tissue damage, which is detrimental to the process of bone repair. In comparison to healthy tissues, inflamed tissues exhibit ROS concentrations that are 10–100 times higher (Liu et al., 2016). Consequently, the development of a scaffold that is responsive to ROS for the purpose of regulating drug release in inflammatory sites and other afflicted tissues represents a promising approach for enhancing bone repair (Mura et al., 2013).

A critically-sized bone defect refers to a clinical situation wherein bone loss or removal occurs as a result of trauma, infection, tumor, or other factors, and is unable to undergo spontaneous healing (Huang et al., 2022). In such circumstances, the defect lacks the ability to self-repair and necessitates external interventions. A recent investigation has documented a study on a polycation that exhibits compatibility with the Layer-by-Layer (LBL) technique and is exclusively degraded by ROS produced by cells. When the concentrations of ROS increase in the surrounding environment, the thioketal-based polymers containing a scaffold structure can be activated and broken down by physiological levels of ROS. Additionally, these polymers enable the controlled release of therapeutic BMP-2 upon oxidation. The findings of this study suggest a direct correlation between ROS-responsive scaffolds and the promotion of bone growth in critically-sized bone defects (Martin et al., 2021).

In the context of pathological tissues, the maintenance of a stable structure by the ROS response system assumes critical importance. Nevertheless, the development of a ROS-responsive system that operates optimally under specific conditions presents a persistent challenge owing to the intricate and heterogeneous *in vivo* microenvironment. Despite continuous endeavors, there persist unresolved fundamental concerns that impede its attainment of perfection.

### 2.3 Enzyme stimuli

Enzymes have found application in the realm of nanotechnology, particularly in the development of nano-drug carriers, owing to their distinctive biological targeting and catalytic attributes. In the context of lesion tissue, enzyme levels undergo alterations within the local microenvironment as a consequence of injury and inflammation. By employing this approach, enzymes can be directed towards specific biochemical signals within the area of bone defect, facilitating the regulation of active ingredient release.

**FIGURE 5:**
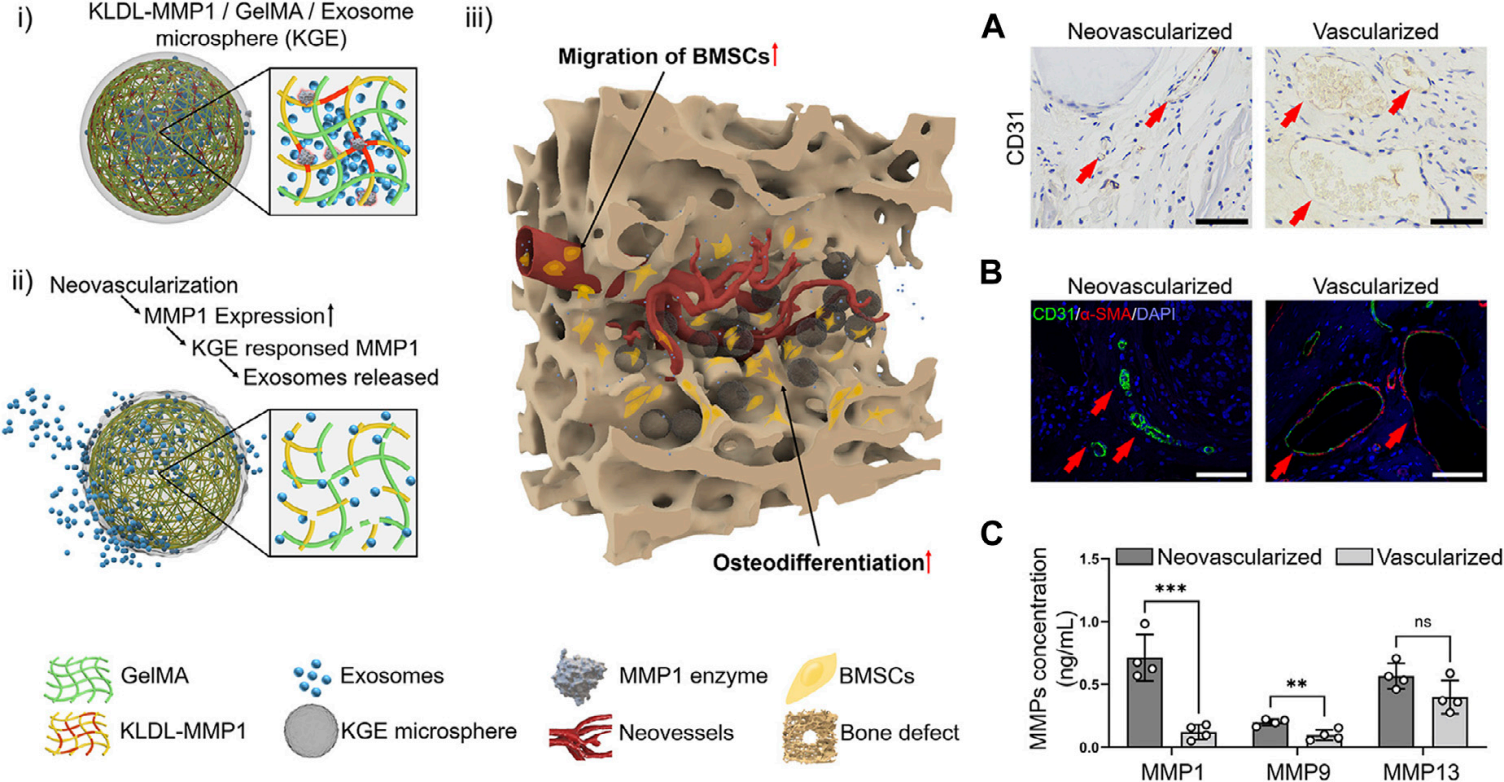
Structural design of enzyme-responsive KGE microspheres. **(i)** KGE microspheres encapsulated Exos before enzymatic hydrolysis. **(ii)** MMP1 degrades KGE, releasing Exos after injection. **(iii)** Through neovessels, Exos diffuse into bone defects to promote BMSCs migration and osteodifferentiation. **(A–C)** The MMPs expression in neovascularized and vascularized bone tissues. **(A)** CD31 immunohistochemical staining of SD rat skull defect on day 14, scale = 100 μm. **(B)** CD31/α-SMA Immunofluorescent staining of SD rat skull defect on day 14, scale = 100 μm. **(C)** Detection of MMPs concentration by ELISA, ∗∗∗: *p* < 0.001, ∗∗: *p* < 0.01, ns: *p* > 0.05. Reproduced from (Jia et al., 2023) with permission. Copyright 2023 Elsevier.

Neovascularized bone, for example, expresses high levels of matrix metalloproteinase-1 (MMP1) (Quintero-Fabián et al., 2019). MMP1 can degrade extracellular matrix proteins by cleaving specific amino acid sequences, which can promote the migration of vascular endothelial cells by decomposing the extracellular matrix (Quintero-Fabián et al., 2019). The KLDL-MMP1 (Ac-KLDLKLDLVPMSMRGGKLDLKLDL-CONH2) peptides were synthesized by the researchers, as they can be degraded by MMP1. To develop a microfluidic chip, the researchers utilized an injectable MMP1-sensitive hydrogel microsphere (KGE), which was created by combining self-assembling peptide (KLDL-MMP1), gelatin methacryloyl (GelMA), and bone marrow mesenchymal stromal cell-derived exosomes (BMSC-Exos). The Exo-release material, which is sensitive to enzymes, exhibits a specific response and degradation towards MMP1 originating from neovascularization during the angiogenesis phase subsequent to bone injury. This degradation process facilitates the release of exosomes within scaffolds, thereby facilitating the recruitment of cells for the purpose of bone defect repair (Yang et al., 2023) (Figure 5).

A novel enzyme-responsive scaffold has been developed utilizing glucose oxidase (GOD), an enzyme capable of selectively catalyzing the degradation of glucose. In individuals with diabetes, the process of osteogenesis is frequently hindered due to the presence of elevated glucose levels in the body, which in turn leads to inflammation that inhibits osteogenesis. In a high-glucose environment, glucose can undergo specific catalysis by glucose oxidase, resulting in the production of gluconic acid. Consequently, the researchers incorporated glucose oxidase into the nanofiber scaffold to construct a glucose oxidase responsive scaffold. As the glucose concentration increased, the nanofiber scaffolds gradually expanded, leading to the subsequent release of dexamethasone (DEX), which possesses anti-inflammatory properties and promotes bone formation. Thus, these glucose-sensitive nanofiber scaffolds present a promising therapeutic approach for individuals with diabetes and alveolar bone defects (Jia et al., 2023).

Despite the extensive development of enzymatic reaction systems, they still possess several drawbacks within an academic context. One such limitation pertains to the variability in enzyme expression levels observed among patients, thereby raising concerns regarding the adequacy of enzyme expression within the target population. Additionally, the lack of specificity poses another challenge, as different types of matrix metalloproteinases (MMPs) may exhibit cross-reactivity. For example, all of MMP1, MMP8, and MMP13 can cleave glycine–isoleucine or glycine–leucine bond (Williams and Olsen, 2009). These limitations ultimately impede the progress of enzyme-reactive scaffolds in the field of bone engineering.

## 3 Conclusion and discussion

Stimuli-responsive scaffolds have emerged as a promising class of intelligent biomaterials in recent years. The advantages and limitations of responsive scaffold categories are shown in Table 2. They possess the ability to detect various physical stimuli, including light, temperature, electric field, magnetic field, and ultrasound, as well as chemical stimuli such as pH and redox response, and enzyme stimuli. Upon encountering specific stimuli, these scaffolds facilitate cell adhesion, migration proliferation, and differentiation. Consequently, they hold great potential for the repair of bone defects. Despite the notable progress made in biomaterial advancements for bone tissue engineering over the past few decades, there remains a considerable amount of work to be done, particularly in three specific areas that warrant further investigation in the future: 1) Responsive scaffolds for bone tissue engineering must possess specific biocompatibility and exhibit targeted responses to particular stimuli. However, achieving a singular response is challenging due to the intricate nature of the human physiological environment and the diverse conditions found at injury sites. Consequently, the development of multi-response scaffolds is gradually gaining momentum as a means to attain optimal therapeutic outcomes; 2) Further in-vivo experiments are necessary to ascertain the interactions between biomaterials and the local microenvironment. The implantation of biomaterials can induce substantial alterations in the microenvironment, thereby exerting a significant influence on osteogenesis. Consequently, it is imperative to continuously monitor the dynamic changes of substances within the body; 3) Furthermore, the challenges pertaining to the precision and specificity of responsive tissue engineering scaffolds persist. The accurate identification of the lesion site and the implementation of targeted responses necessitate additional attention and research. Continued advances in bone tissue engineering are anticipated to facilitate the rapid development of stimuli-responsive scaffolds, offering additional treatment options for the clinical management of bone defects, and ultimately influencing clinical outcomes.

**TABLE 2:** The advantages and limitations of responsive scaffold categories.

Scaffold categories	Advantages	Limitations
Temperature-responsive	Controllable and easy to operate	Temperature ranges are limited
Light-responsive	Little harm to the human body; No direct contact with the lesion area	Penetration depths are either too shallow or too deep; The biological safety of light-sensitive materials need to evaluate
Electric-responsive	Responds quickly and with strong sensitivity	Further investigation is required to examine the alterations in electric field intensity and frequency within organisms
Mechanical-responsive	Sensitive and quick to react	Potential toxicity resulting from the use of crosslinking agents
Magnetic-responsive	Controllable from an external source	The diffusion rate of magnetic particles is high; There exists a potential for toxicity
Ultrasound-responsive	The system can be controlled externally	The controllability of penetration depth is limited; It generates a thermal effect
pH-responsive	Utilizing the distinctions between diseased and normal tissue	Limited range of variation; Low sensitivity
Redox-responsive	Utilizing the distinctions between diseased and normal tissue	The sensitivity is relatively low
Enzyme-responsive	Distinctive biological targeting and catalytic attributes	Different types of enzymes may exhibit cross-reactivity
